# Exploring Locus-of-Hope: Relational Tendencies, Self-Esteem, Attachment, and Gender

**DOI:** 10.3390/bs11090120

**Published:** 2021-09-03

**Authors:** Sereena Dargan, Kristi Baerg MacDonald, Julie Aitken Schermer

**Affiliations:** 1 Department of Psychology, The University of Western Ontario, London, ON N6A 5C2, Canada; sdargan@uwo.ca (S.D.); kmacd252@uwo.ca (K.B.M.); 2 Departments of Management and Organizational Studies, The University of Western Ontario, London, ON N6A 5C2, Canada

**Keywords:** hope, self-esteem, attachment, gender, locus of hope

## Abstract

Background: As little research has been devoted to examining associations between the four locus-of-hope dimensions (internal, external—peer, external—family, and external—spiritual) and individual differences, the current study explores the correlations with individual-level individualist and collectivist relational tendencies, self-esteem, insecure attachment, and gender within a culturally diverse sample of university undergraduate students. Methods: questionnaires were completed by a culturally diverse sample of undergraduate students measuring locus-of-hope, individualist and collectivist relational tendencies, self-esteem, insecure attachment, and gender. Results: State and trait locus-of-hope were significantly correlated. Individualism showed positive correlations with internal and external—family locus-of-hope. Collectivism positively correlated with internal locus-of-hope and the three external locus-of-hope dimensions. Internal locus-of-hope was significantly predicted by self-esteem, relational self-esteem, individualism, and collectivism. External—spiritual locus-of-hope was not significantly predicted by the variables. External—family locus-of-hope was significantly predicted by relational self-esteem and collectivism and external—peer locus-of hope was significantly predicted by relational self-esteem, collectivism, and avoidant attachment style. No significant gender differences in locus-of-hope were found. Conclusions: The results provide further understanding about the construct of locus-of-hope and provide a foundation for future research to continue exploring the role of locus-of-hope in the development and expression of self-esteem and attachment profiles.

## 1. Introduction

Currently, within psychology, a widely accepted definition of hope comes from Snyder et al.’s Hope Theory [1], in which hope is defined as a cognitive set involving an individual’s motivation and ability to pursue goals. Since its conceptualization, measures of both trait and state hope [2] have been employed across a variety of research contexts. Results from such studies have demonstrated positive associations between hope and several outcomes, including positive coping strategies, life satisfaction, self-esteem, and success in academics, sports, and the workplace [3,4,5,6,7], highlighting the importance of hope within the context of positive psychology research and intervention.

More recently, Snyder et al.’s Hope Theory [1] was extended to include the locus-of-hope dimensions. The extension was put forth on the notion that relational or interpersonal processes, which had been unaccounted for in previous hope literature, might be critical to gaining an understanding of how hope operates across individuals [8]. Although the new perspective, of an external locus-of-hope, has revitalized hope research, relatively little is understood on how locus-of-hope relates to other relevant individual difference variables. The present study aimed to bridge this gap by examining associations between locus-of-hope and a number of predictor variables, specifically individualist and collectivist relational tendencies, self-esteem, insecure attachment, and gender, in an attempt to provide a better understanding of how locus-of-hope is experienced.

### 1.1. Hope Theory and Locus-of-Hope

Snyder [9] described hope as a cognitive process consisting of three components. The first is the presence of clearly conceptualized and subjectively valuable goals. The second, agency thinking, describes levels of motivation and determination to achieve goals. The third component, pathway thinking, refers to the perceived ability to plan for goal achievement. Although Hope Theory and its corresponding psychometric measures [1,2] have been studied, with some results demonstrating replication and therefore support for the theory and the measures of the construct, there have been criticisms for the emphasis Hope Theory places on the individual in achieving agency and pathway thinking, while discounting the role of external relational processes. In fact, other qualitative analyses have pointed to the influence of external agents, such as family and a higher power, in the experience of hope [10]. In light of the above limitation, Bernardo [8] extended hope theory by proposing the addition of the locus-of-hope dimensions. The extension allows agency and pathway thinking to be achieved not only by the individual (now termed the internal locus-of-hope), but also through relationships with members of family (external—family locus-of-hope), peers (external—peer locus-of-hope), and spirituality (external—spiritual locus-of-hope). Together, the internal and three additional external dimensions are therefore used to denote the locus by which agency thinking, pathway thinking, and resulting levels of hope are achieved.

The trait Locus-of-Hope Scale [10] was developed by retaining the eight original items of Snyder’s Adult Dispositional Hope Scale (ADHS; [1]) as a measure of internal locus-of-hope. An additional 32 items were created to assess the three external dimensions. Since its publication, the trait Locus-of-Hope Scale has been used in a small variety of studies. For instance, associations have been found between the locus-of-hope dimensions and select social axioms, collaborative learning strategies, coping strategies, help-seeking intentions, as well as outcomes related to self-esteem and life satisfaction [11,12,13,14]. However, despite the effort to introduce the concept into the literature, the amount of data produced and the resulting understanding of locus-of-hope remain low.

### 1.2. Locus-of-Hope and Individualism-Collectivism

In the extension of hope theory, Bernardo [8] reasoned that internal locus-of-hope reflected a disjoint model of agency, common to westernized, individualist cultures and societies. Disjoint agency assumes positive actions originate from and are expressed in terms of the goals and preferences of the individual [15]. Conjoint or endogenous social models of agency allow for the influence of interpersonal expectations and social roles in expressing positive actions and goal pursuits [16]. These latter models are common within Eastern cultures endorsing collectivist tendencies and were thought to be better reflected by the external dimensions of hope. This hypothesis was tested in a sample of students from a university in Manila, Philippines. Consistent with predictions, the four locus-of-hope dimensions were shown to be distinct and significant components to the conceptualization of hope, with individualism significantly and exclusively predicting internal locus-of-hope, and collectivism predicting the three external locus-of-hope dimensions [8].

Subsequent studies have further validated associations between locus-of-hope and individualist and collectivist relational tendencies through correlations with independent and interdependent self-construals (how the self is perceived in relation to others), respectively [17]. Importantly, Du and King [17] noted that their results might partially reflect the prevailing collectivist norms within the population from which participants were drawn, particularly in terms of external locus-of-hope cognitions. In other words, collectivism, and its associated models of agency and interdependence, might not be as significant a component to hope cognitions within cultures and societies where collectivist relational tendencies are not so strongly endorsed. That said, there remains a need to cross-culturally validate associations between locus-of-hope and these relational tendencies within samples endorsing differing degrees of collectivist and individualist values. Producing results consistent with Bernardo’s predictions [8] would further support the locus-of-hope construct and its theoretical foundations across populations. The current study therefore examined associations between locus-of-hope and individual-level individualist and collectivist relational tendencies in a sample population identifying with a variety of cultural backgrounds.

### 1.3. Locus-of-Hope and Self-Esteem

In addition to re-examining the importance of individualist and collectivist relational tendencies to the locus-of-hope construct, there is also a need to discern how other individual difference variables relate to the construct of locus-of-hope. For instance, the relevance of trait hope is greatly attributed to its associations with measures of subjective well-being and life satisfaction [6,9,18]. However, very little is understood about how the external locus-of-hope dimensions relate to similar measures, making its utility difficult to extrapolate. In a study aimed at addressing this gap, Du and King [17] examined internal and external locus-of-hope with three measures of psychological adjustment: life satisfaction, personal self-esteem, and collective self-esteem. Results show that the internal locus-of-hope scale had positive correlations with life satisfaction and the two self-esteem scales, replicating past findings. For the external—family locus-of-hope scale and the external—peer locus-of-hope scale, positive correlations were also found between life satisfaction and self-esteem (both personal and collective). In contrast, the spiritual locus-of-hope scale had non-significant and negative correlations with life satisfaction scores and both personal and collective self-esteem scales. These results suggest that not all external locus-of-hope dimensions relate to other individual difference measures in the same manner, suggesting that further research is required.

In a subsequent study, focusing on life satisfaction along with personal and relational self-esteem, internal locus-of-hope, external—family, and external—peer locus-of-hope scales demonstrated positive correlations with scale scores on life satisfaction and relational self-esteem scales [14]. The external—spiritual locus-of-hope scores did not correlate significantly with the two self-esteem scales, replicating the results of Du and King [17]. In contrast, the external—spiritual locus-of-hope has a significant positive association with life satisfaction. In light of the conflicting results regarding external locus-of-hope dimensions and adjustment, the current study did not make explicit a priori hypotheses, but aims to build upon these past findings by continuing investigations correlating locus-of-hope scales with self-report self-esteem.

### 1.4. Locus-of-Hope and Attachment

The current study also sought to broaden knowledge on locus-of-hope by examining associations with adult attachment. According to Ainsworth and Bowlby’s attachment framework [19], three primary attachment styles have been identified based on child-caregiver relationships. Secure attachment results from goals and needs for security and proximity being appropriately satisfied by caregivers, whereas attachment anxiety and attachment avoidance (two types of insecure attachment) ensue when needs are inconsistently, or rarely, met [19]. These early attachment relationships are fundamental to the development of positive (or negative) models of the self and close others. As such, the resulting attachment styles often persist throughout adulthood, reflected by distinct sets of attachment-related goals and characteristics [20,21].

The relevance of hope to attachment relationships was introduced in Snyder’s earlier work [22] suggesting that secure early attachment forms the foundation for goal-directed thought (agency and pathway thinking) and therefore contributes to overall levels of hopefulness in adulthood. Meta-analytic results have supported this suggestion, demonstrating positive correlations between trait hope and secure attachment and negative correlations between hope and insecure attachment [23]. While research has yet to address how Bernardo’s locus-of-hope dimensions [8] fit into Snyder’s proposed attachment-hope dynamic [22], it is likely that attachment anxiety and attachment avoidance are differentially associated with external locus-of-hope based on the distinct goals and characteristic attachment profiles. For instance, adult attachment avoidance reflects a negative working model of close others. Characteristics include fears of interpersonal intimacy and excessive self-reliance, while attachment goals are geared towards achieving distance from others as a means of avoiding vulnerability and dependency [24]. Here, negative perceptions of the agency and pathway abilities of close others are more likely. In contrast, individuals high in attachment anxiety adopt negative working models of the self and might be expressed as a need for excessive responsiveness and approval from partners. Attachment goals here consist of satisfying unfulfilled security needs and fears of abandonment through strategies of seeking extreme closeness or dependency on partners [25]. Such characteristics would likely be positively associated with external agency and pathway thinking, as satisfying these needs are contingent upon perceptions of close others as being capable and available. To test these predictions, the current study examined correlations between attachment avoidance, attachment anxiety, and locus-of-hope.

### 1.5. Locus-of-Hope and Gender

Gender differences in Snyder’s internal trait hope [1] have been noted in some investigations, with men exhibiting higher trait hope compared with women [26,27,28]. However, prior to the current study, there have been no investigations, to our knowledge, on potential gender differences in locus-of-hope dimensions. Therefore, within studies citing gender differences, it might be the case that women simply differed in their experience of hope, utilizing more external over internal loci, rather than experiencing less hope overall. Indirect support for this idea can be found in research demonstrating that women engage in higher levels of collectivist relational tendencies [29] and allocentrism [30] in addition to placing a higher value on assisting others with their goals [31]. These findings suggest that women may perceive the desires, expectations, motivations, and abilities of close others as having a stronger influence over how their personal goals can be pursued and achieved. To explore this idea, the present study examined potential gender differences in locus-of-hope and assessed if gender significantly predicted variance in the locus-of-hope scales.

### 1.6. Present Study

Based on the above review, it is clear that there exists a comparatively small amount of research on, and therefore understanding of, locus-of-hope and its relation to individual difference variables. In light of this, the present study was designed to provide additional insights into the construct to aid in the understanding of cognitive hope, its use, and its significance across individuals. To achieve this, the present study had three aims. First, to assess whether the four locus-of-hope dimensions demonstrate expected correlations with individual-level individualism and collectivism within a sample with diverse cultural backgrounds. In line with Bernardo’s predictions [8], individualism was hypothesized to show positive correlations with internal locus-of-hope, and collectivism to have positive correlations with the three external locus-of-hope dimensions. Second, to address the lack of consensus on the relationship between locus-of-hope and well-being, this study examines the correlations between locus-of-hope and personal and relational self-esteem. Consistent with results of previous research, internal, external—family, and external—peer locus-of-hope scores are predicted to show positive correlations, and external—spiritual locus-of-hope negative correlations, with both measures of self-esteem [17]. The third aim of the study was to extend our current knowledge of how locus-of-hope operates across individuals. Particularly, how locus-of-hope is associated with insecure attachment and gender. Attachment avoidance was hypothesized to show negative correlations with internal and external locus-of-hope dimensions. Attachment anxiety, on the other hand, is expected to show negative correlations with internal locus-of-hope and positive associations with external locus-of-hope. Additionally, women, who tend to demonstrate more collectivist relational tendencies [29], were hypothesized to exhibit higher external and men higher internal locus-of-hope. Finally, for replication purposes, as well as to contribute to the validity of the Pilot State Locus-of-Hope Scale [32,33], it was predicted that state and trait locus-of-hope scores would be positively correlated. In order to examine how the individual difference variables relate to the dimensions of locus-of-hope, the present study first examines the zero-order correlations between the scales and then uses the variables as predictors to assess how each variable independently contributes to the variance in each locus-of-hope dimension.

## 2. Materials and Methods

### 2.1. Participants

Participants consisted of 549 university students (280 males, 251 females, and 18 missing) enrolled in an undergraduate management and organizational studies class. Participants were compensated with a research credit to fulfill a component of their course. The study was completed individually and online. A total of 120 different cultures or combinations of cultures were reported (see Appendix A). The data are available from the authors.

### 2.2. Instruments

#### 2.2.1. Locus-of-Hope

The 40-item Locus-of-Hope Scale [8] is a self-report measure composed of four subscales, each containing eight items: internal locus-of-hope (e.g., “I energetically pursue my goals.”; coefficient alpha (α) = 0.82 for the present sample), external—peer (e.g., “With the help of my friends, I am confident that I can reach my goals in life.”; for the present sample, α = 0.85), external—family (e.g., “My family finds many ways to help me solve my problems.”; α = 0.89 in this sample), and external—spiritual locus-of-hope (e.g., “If it is God’s will, I will meet the goals that I set for myself.”; α = 0.98 in the present study sample). Each subscale equally assesses both agency and pathway thinking. Participants replied to the items on a Likert-type scale ranging from 1 = definitely false to 4 = definitely true.

#### 2.2.2. State Locus-of-Hope

The short version of the State Locus-of-Hope Scale [32,33] contains 16 items, with four items devoted to assessing each of the four locus-of-hope subscales: internal (α = 0.75 in this sample), external—family (α = 0.84 in this sample), external—peer (α = 0.87 in this sample), and external—spiritual (α = 0.97 in this sample) locus-of-hope. The scale measures participants’ levels of agency and pathway thinking pertaining to currently held goals in the “here and now” (e.g., “My family presently helps me find ways to solve my current problems.”). Participants responded on a four-point Likert-type scale ranging from 1 = definitely false to 4 = definitely true.

#### 2.2.3. Personal Self-Esteem

The 10-item Rosenberg Self-Esteem Scale [34] was used as a measure of personal self-esteem. This scale contains one dimension devoted to assessing participants’ positive (e.g., “On the whole, I am satisfied with myself.”) and negative (e.g., “I certainly feel useless at times.”) views of self-worth and self-acceptance. The scale demonstrates good reliability in the present sample with a Cronbach’s alpha of 0.88. Responses were provided on a four-point Likert-type scale ranging from 1 = strongly agree to 4 = strongly disagree. Low scores on the Rosenberg Self-Esteem Scale indicate higher levels of self-esteem. Therefore, in order to maintain consistency with other measures, self-esteem scores were reverse coded such that higher scores represent greater self-esteem.

#### 2.2.4. Individualism-Collectivism

The Auckland Individualism-Collectivism Scale [35] was used as a self-report measure of individual-level individualism and collectivism. The scale includes 30 items, 16 of which are devoted to a collectivism subscale (e.g., “I enjoy being unique and different from others.”; for this sample, α = 0.77), while the remaining items assess an individualism subscale (e.g., “I discuss job or study-related problems with my parents.”; for this sample, α = 0.88). The items measure individualist and collectivist relational tendencies by describing particular attitudes and behaviors to which participants self-report their frequency on a 6-point Likert-type scale ranging from 1 = never to 6 = always.

#### 2.2.5. Insecure Attachment

The Experiences in Close Relationship Scale—Short Form [36] was included as a measure of participants’ attachment orientations. This scale contains 12 items, six of which assess the dimension of attachment anxiety (e.g., “I get frustrated if romantic partners are not available when I need them.”; for this sample, α = 0.71), while the remaining six assess attachment avoidance (e.g., “I try to avoid getting too close to my partner.”; for this sample, α = 0.68). Responses were made on a seven-point Likert-type scale ranging from 1 = strongly disagree to 7 = strongly agree.

#### 2.2.6. Relational Self-Esteem

The Relational Self-Esteem Scale [37] assessed participants’ relationships with significant others such as family and close friends, the perceived value of those relationships (e.g., “I am proud of my family.”), as well as one’s own value within them (e.g., “I can help my friends a lot.”). The unidimensional construct is assessed with eight items (in the present sample, α = 0.85) and answered on a four-point Likert-type scale ranging from 1 = strongly disagree to 4 = strongly agree.

In addition to the above instruments, participants responded to demographic questions including fill-in-the-blank questions on gender and general cultural identity.

### 2.3. Procedure

Participants completed the online surveys using Qualtrics. Informed consent was obtained. The study received institutional ethics approval.

### 2.4. Statistical Analyses

Descriptive statistics were calculated, followed by the inter-scale correlations (including gender) using SPSS version 24 (IBM, Chicago, IL, USA). All probabilities were two-tailed, with the level of significance set at *p* < 0.01. Missing data were treated using pairwise deletion. Subsequently, four direct entry regression analyses were computed with the study variables predicting internal locus-of-hope, external—spiritual locus-of-hope, external—family locus-of-hope, and external—peer locus-of-hope, respectively. Regression analyses were completed using R version 4.0.2.

## 3. Results

Correlations and descriptive statistics are presented in Table 1.

### 3.1. State and Trait Hope

In line with the first hypothesis, results indicate that scores on the internal trait locus-of-hope subscale were significantly correlated with those of internal state locus-of-hope. Similar results were found for external—peer, external—family, and external—spiritual locus-of-hope, indicating that participants’ levels of state and trait hope were highly inter-correlated.

### 3.2. Individualism-Collectivism and Locus-of-Hope

Individual-level individualism and collectivism, as measured by scores on the Auckland Individualism-Collectivism Scale [35], were both significantly correlated with internal locus-of-hope. Individualism scores were also significantly correlated with scores of external—peer and external—family locus-of-hope; however, correlations between individualism and external—spiritual locus-of-hope were not significant. Collectivism scores were significantly correlated with scores on all three of the external locus-of-hope subscales.

### 3.3. Locus-of-Hope and Self-Esteem

Internal locus-of-hope scores were significantly correlated with both personal and relational self-esteem. External—peer and external—family locus-of-hope scores were also significantly correlated with both personal and relational self-esteem. In contrast, scores between external—spiritual locus-of-hope and self-esteem did not reach significance.

### 3.4. Insecure Attachment and Locus-of-Hope

Scores on the attachment avoidance subscale demonstrated significant negative correlations with both internal and external—family locus-of-hope scores. Correlations between attachment avoidance and external—peer and external—spiritual locus-of-hope were nonsignificant. In contrast, scores on the attachment anxiety subscale were not significantly correlated with internal locus-of-hope. In terms of the external locus-of-hope subscales, attachment anxiety scores showed significant positive correlations with only external—peer locus-of-hope.

### 3.5. Gender and Locus-of-Hope

Point-biserial correlations between gender and internal locus-of-hope were nonsignificant. Similarly, correlations between gender and external—peer, external—family, and external—spiritual locus-of-hope were also nonsignificant. Therefore, contrary to the hypotheses, the results demonstrate no significant gender differences in the use of internal and external trait locus-of-hope.

### 3.6. Regression Analyses

Table 2 lists the regression results from a direct entry regression analysis to predict internal locus-of-hope. The overall model was significant (*F*(7, 459) = 59.08, *p* < 0.001), accounting for 47% of the variance. Internal locus-of-hope was significantly predicted by self-esteem, relational self-esteem, individualism, and collectivism.

Table 3 provides the results of a direct entry regression analysis predicting external—spiritual locus-of-hope. Although significant (*F*(7, 460) = 2.87, *p* = 0.006), this model only accounted for 4% of the variance and none of the study variables uniquely and significantly predicted external—spiritual locus-of-hope.

Table 4 presents the results of a direct entry regression analysis predicting external—family locus-of-hope. In contrast with the external—spiritual locus-of-hope results, 35% of the variance in external—family locus-of-hope was significantly accounted for by the study variables (*F*(7, 455) = 35.24, *p* < 0.001). Specifically, external—family locus-of-hope was significantly predicted by relational self-esteem and collectivism.

Table 5 presents the results of a direct entry regression model using the study variables to predict external—peer locus-of-hope. The study variables were found to account for 23% of the variance in external—peer locus-of-hope and the overall model was significant (*F*(7, 456) = 19.69, *p* < 0.001). For individual variables, external—peer locus-of-hope was significantly predicted by relational self-esteem, collectivism, and avoidant attachment style.

Overall, external—spiritual locus-of-hope was not well predicted by the analyzed variables, in contrast to internal, external—family, and external—peer, which all had above 23% of the variance predicted by the study variables. Among these three locus-of-hope constructs, relational self-esteem was an important predictor for all of them, when examining the 95% confidence intervals. Personal self-esteem was significant only for internal locus-of-hope. Consistent with study expectations, individualism was predictive of internal locus-of-hope, and collectivism predictive of external—family and external—peer. Somewhat surprising is that collectivism also significantly predicted internal locus-of-hope.

## 4. Discussion

The purpose of the current study was to explore locus-of-hope in relation to individual-level individualism and collectivism, self-esteem, insecure attachment, and gender within a sample with diverse cultural backgrounds. In particular, this study examines the relatively new construct of sources of external locus-of-hope in addition to the traditional internal locus-of-hope dimension.

### 4.1. State and Trait Locus-of-Hope

In line with the prediction that state hope fluctuates within the bounds of trait or dispositional hope [2], it was hypothesized that scores from measures of state and trait locus-of-hope would be highly inter-correlated. Indeed, results did show statistically strong inter-correlations between the state and trait locus-of-hope dimensions, lending support to the criterion validity of the recently developed State Locus-of-Hope Scale [32,33].

### 4.2. Individualism-Collectivism and Locus-of-Hope

The first two hypotheses were confirmed, as positive correlations between individualism and internal locus-of-hope as well as collectivism and the three dimensions of external locus-of-hope were found to be statistically significant. Individualist relational tendencies demonstrated greater magnitude correlations with internal rather than external locus-of-hope, while collectivist relational tendencies showed greater magnitude correlations with the external rather than internal locus-of-hope dimensions (with the exception of external—spiritual hope, which demonstrated only small correlations with collectivism). In addition, the regression models demonstrated that internal locus-of-hope was significantly predicted by both individualism and collectivism, with a stronger beta-weight for individualism. External—family locus-of-hope and external—peer locus-of-hope were both significantly predicted by collectivism and not for individualism. For the external—spiritual locus-of-hope scale, collectivism had a positive beta-weight, but the value did not reach significance. These findings provide some support to the theory behind Bernardo’s additional external locus-of-hope dimensions [8], in which individualist relational tendencies are related to internal locus-of-hope, while collectivist tendencies are best reflected by external locus-of-hope for at least the peer and family sources.

### 4.3. Locus-of-Hope and Self-Esteem

In the present study, self-esteem was assessed by examining both personal self-esteem, assessing self-worth, and relational self-esteem, assessing pride with friends and family associations. The hypothesized positive correlations between internal locus-of-hope and the two measures of self-esteem were supported as the correlations were statistically significant and corroborate the general conclusion that internal locus-of-hope relates to measures of well-being. In particular, when predicting the variance in internal locus-of-hope, both personal and relational self-esteem scales predicted significant independent components of variance. The associations between self-esteem and internal locus-of-hope are well-documented and are likely due to the increases in self-efficacy and optimism that ensue from the adoption of internal agency and pathway thinking [38].

The correlation, and regression weights, between the external—spiritual locus-of-hope and self-esteem did not reach significance for both of the self-esteem scales. These results are in partial agreement with past studies [14,17]. In interpreting these results, it is important to consider the possibility that the degree to which external—spiritual hope contributes to adaptive characteristics is affected by participants’ degree of religious orientation. Indeed, analyses incorporating large-scale, cross-national datasets have shown that the association between religiosity and well-being is significantly influenced by country-wide levels of religiosity and religious attitude [39,40]. Future research may wish to explore the potential for both country-wide and individual-level religiosity to mediate associations between external—spiritual hope and aspects of well-being and adjustment.

Due to conflicting reports in the previous literature, no specific predictions were made for the external—peer and external—family locus-of-hope dimensions in relation to self-esteem. That said, the results of the regression analyses in the current study indicate that both external—peer and external—family locus-of-hope were associated with higher relational self-esteem, similar to the findings of Du and King [17], but the predictor weights for personal self-esteem were nonsignificant. Importantly, the current results contribute to a growing view that relying on family or close friends for agency and pathway thinking throughout goal pursuit is associated with higher levels of self-worth and self-acceptance through associations with friends and family. In essence, these locus-of-hope dimensions are associated with higher perceptions of the value of close others.

In contrast to the internal locus-of-hope, little is understood about how external locus-of-hope is related to self-esteem. While external locus-of-hope dimensions likely involve the positive efficacy evaluations that follow from advancement towards a goal state, as well as perceptions of close others as available and willing to offer support for the advancement of personal goals, understanding the specifics of such mechanisms will be up to future investigations. Overall, internal trait hope has long been recognized as a protective factor of individual quality of life and general well-being within both clinical and non-clinical populations [41,42,43,44]. The results of the current study suggest that the external dimensions of hope, in particular external—family and external—peer, show promise as protective factors through their association with relational self-esteem and warrant further exploration for general and clinical applications.

### 4.4. Insecure Attachment

In partial agreement with the hypotheses, attachment avoidance was significantly and negatively associated with both internal and external—family locus-of-hope. The former result is consistent with Snyder’s theory of hope and attachment [22] and has been frequently reported in past research [23]. The latter result is consistent with characterizations of the avoidant attachment profile. Particularly, individuals with negative models of close others would be expected to avoid the emotional intimacy and dependence associated with sharing personal goals and relying on others for their achievement [45]. Such an association is also intuitive from the perspective of early attachment theory because the pattern of behaviors that typically result in avoidant attachment towards caregivers would likely go hand-in-hand with the poor development of an external—family locus-of-hope. Future research might demonstrate this pattern of relationships empirically by investigating the role of secure and insecure early attachment in predicting external—family locus-of-hope in children.

Correlations, and regression weights, between attachment anxiety and internal locus-of-hope did not reach significance. This was somewhat surprising considering the amount of past research supporting negative associations between both insecure attachment styles and levels of hope [23]. Attachment anxiety was significantly and positively correlated with external—peer locus-of-hope but, when entered into a regression model, attachment anxiety did not significantly predict external—peer locus-of-hope. Therefore, the present data partially support the idea that the hope that anxiously attached individuals have is largely unrelated to perceptions of their own ability, but rather to that of their social network or peers. Additionally, these results partially support theoretical conceptualizations of anxiously attached individuals as desiring to uphold close relationships with significant others who are perceived as involved in the attainment of personal needs and goals [46] and may reflect the need of young adults (undergraduate students) to rely on relationships with peers, close friends, and romantic partners more than parents and family [47,48]. These results, although correlational in nature, may have important implications in attachment research, particularly in understanding the role of external locus-of-hope in both the potential development of healthy attachment and the maintenance of insecure attachment.

### 4.5. Gender

The hypotheses that males would report higher internal locus-of-hope, and women would report higher external locus-of-hope, were not statistically supported. The only significant result found for gender was a small negative correlation with internal state locus-of-hope, indicating that males in the sample had slightly higher internal hope scores, but it should be noted that the gender beta-weight in the direct entry regression model predicting internal locus-of-hope was not significant. The absence of significant results is consistent with studies reporting no significant gender differences on other variables that are conceptually similar to hope as well as the agency and pathway thinking constructs, such as achievement goals, self-efficacy, or motivation [49,50,51]. The results are also consistent with the findings by Snyder et al. [1], which indicate that, despite what might be predicted by traditional Westernized gender roles, men and women generate internal agency and pathway thinking to equal degrees in the process of goal pursuit. Other variables closely related to external locus-of-hope have yet to be identified by research. However, while the lack of significant gender differences opposes the hypothesis, it is in line with observations that men and women do not consistently exhibit significant differences in the degrees to which they perceive external support from peers and family [52,53]. In the case of male and female caregivers, Rodríguez-Madrid et al. [54] also found no significant gender differences in the amount or type of support received from support networks. These results, along with the current findings, suggest that while females’ self-construals (and their associated attitudes and behaviors) tend to be more relational or interdependent in nature, this does not lead to perceptions of family, friends, or spirituality as having any more of a role in the attainment of personal goals. Importantly, these results provide the first evidence demonstrating the stability of the external locus-of-hope dimensions across gender.

## 5. General Limitations and Conclusions

The present study also included a number of limitations. For example, although participants in the sample identified with a wide variety of cultures that vary in their estimated country-wide rankings of individualism and collectivism, the distribution of the sample was somewhat unbalanced. China (a strongly collectivist culture) accounted for the largest portion of responses (19.4%; see Appendix A) even though the study was conducted in Canada. Additionally, the general cultural identity of a large number of participants went unidentified.

In addition, the cross-sectional and correlational nature of the data prohibits any inference of causality or directionality between the variables. Future research involving longitudinal or experimental design is required to properly understand whether high self-esteem is a trait that fosters locus-of-hope, or vice versa. Similar investigations would benefit the study of locus-of-hope and attachment, as such an understanding might prove important not only theoretically, but also practically, in the application of hope intervention programs [55,56]. In addition, lifestyle and health variables were not included in the present study. How the different sources of hope influence individuals’ health and well-being is an area requiring future research.

In conclusion, the study showed that the ways in which individuals achieve a sense of hope go beyond the original conceptualization in which agency and pathway thinking are required to come from the individual to whom the goals belong. Rather, the current study validates findings that hope can also be achieved externally, through the motivation and abilities of close others, and that this means of achieving hope is more common among individuals exhibiting collectivist or interdependent relational tendencies. Therefore, this four-dimensional conceptualization of hope represents a more culturally diverse approach to the measurement of cognitive hope across populations. In addition, the results of this study highlight the potential of locus-of-hope in contributing to positive outcomes, such as personal and relational self-esteem, and provides a basis for future investigations on the role of locus-of-hope in the formation of secure and insecure attachment. There remains a long way to go in understanding how individual differences impact the experience and expression of hope as well as the outcomes, both positive and negative, that hope might provide. Fortunately, the adoption and validation of such an inclusive measure represent a strong step in that direction and provide a foundation on which future research may begin properly addressing such questions.

That said, the impact of cognitive hope goes far beyond subjective measures of self-esteem and, until recently, its array of positive and protective outcomes was understood as being the result of only the individual’s motivations and abilities. The recent addition of the external dimensions raises questions about the extent to and mechanisms by which external—peer, external—family, and external—spiritual hope similarly contribute to positive personal and relational outcomes. These questions provide opportunities to the field of positive psychology to further explore the role of the additional dimensions in the development of life satisfaction and well-being, beyond what can be explained by the internal locus.

## Figures and Tables

**Table 1:** Descriptive Statistics and Correlations of Variables of Interest.

Measures	Gender	2	3	4	5	6	7	8	9	10	11	12	13	14	15
2. Internal LOH	−0.08	--													
3. Ext. spirit LOH	−0.07	0.10	--												
4. Ext. fam LOH	0.02	0.47 *	0.20 *	--											
5. Ext. peer LOH	−0.02	0.39 *	0.15 *	0.38 *	--										
6. PSE	−0.13 *	0.49 *	−0.04	0.30 *	0.14 *	--									
7. Relational SE	−0.09	0.50 *	0.11	0.49 *	0.28 *	0.51 *	--								
8. INDI	−0.20 *	0.59 *	0.04	0.29 *	0.15	0.38 *	0.46 *	--							
9. COL	0.08	0.36 *	0.14 *	0.48 *	0.41 *	0.13 *	0.41 *	0.37 *	--						
10. ANX	0.01	−0.02	0.05	−0.02	0.12 *	−0.27 *	−0.09	0.05	0.11 *	--					
11. AVD	0.11 *	−0.19 *	−0.04	−0.22 *	−0.03	−0.25 *	−0.28 *	−0.12 *	−0.12 *	0.14 *	--				
12. Internal state LOH	−0.12 *	0.71 *	0.12 *	0.38 *	0.29 *	0.39 *	0.47 *	0.47 *	0.31 *	−0.01	−0.15 *	--			
13. Ext. spirit state LOH	−0.08	0.11	0.95 *	0.20 *	0.16 *	−0.03	0.11	0.04	0.14 *	0.08	−0.03	0.14 *	--		
14. Ext. fam state LOH	−0.03	0.45 *	0.19 *	0.80 *	0.33 *	0.31 *	0.52 *	0.29 *	0.42 *	−0.01	−0.21 *	0.47 *	0.20 *	--	
15. Ext. peer state LOH	−0.05	0.33 *	0.09	0.31 *	0.73 *	0.18 *	0.35 *	0.18 *	0.39 *	0.08	−0.03	0.38 *	0.13 *	0.41 *	
*Mean*		25.04	17.41	24.97	22.19	21.32	26.74	63.04	65.02	24.91	20.22	12.26	8.73	12.46	11.05
*SD*		3.30	7.84	4.12	3.83	5.52	3.55	10.19	8.87	6.23	5.61	1.91	4.05	2.25	2.42

*Notes:* Internal LOH, internal locus-of-hope; Ext. spirit LOH, external—spiritual locus-of-hope; Ext. fam LOH, external—family locus-of-hope; Ext. peer LOH, external—peer locus-of-hope (Bernardo, 2010). PSE, personal self-esteem (Rosenberg, 1965). Relational SE, relational self-esteem (Du, King, and Chi, 2012). ANX, attachment anxiety; AVD, attachment avoidance (Wei et al., 2007). INDI, individualism; COL, collectivism (Shulruf, Hattie, and Dixon, 2007). Internal state LOH, internal state locus-of-hope, Ext. spirit state LOH, external—spiritual state locus-of-hope; Ext. fam state LOH, external—family state locus-of-hope; Ext. peer state LOH, external—peer state locus-of-hope (Bernardo and Estrellado, 2014; Bernardo and Mendoza, 2020); * *p* < 0.01.

**Table 2:** Regression Coefficients of Study Variables on Internal Locus-of-Hope.

Variable	*b*	*SE*	*β*	95% CI
(Intercept)	−0.93	1.37	0.00	[−2.69, 2.69]
Gender^ a^	0.20	0.24	0.03	[−0.44, 0.50]
Self-esteem	0.16 **	0.02	0.26	[0.21, 0.31]
Relational self-esteem	0.14 **	0.04	0.15	[0.07, 0.23]
Individualism	0.12 **	0.01	0.38	[0.35, 0.41]
Collectivism	0.04 *	0.01	0.10	[0.08, 0.13]
Anxious Attachment	0.02	0.03	0.03	[−0.02, 0.08]
Avoidant Attachment	−0.06	0.03	−0.09	[−0.15, −0.04]

*Notes: b*, Unstandardized regression coefficient; *SE*, Standard error; *β*, standardized regression coefficient; CI, Confidence interval of standardized regression coefficient. ^a^ Women = 1, men = 2, * *p* < 0.01, ** *p* < 0.001.

**Table 3:** Regression Coefficients of Study Variables on External—Spiritual Locus-of-Hope.

Variable	*b*	*SE*	*β*	95% CI
(Intercept)	0.74	4.42	0.00	[−8.68, 8.68]
Gender^ a^	−1.52	0.78	−0.10	[−1.62, 1.43]
Self-esteem	−0.13	0.08	−0.09	[−0.25, 0.07]
Relational self-esteem	0.29	0.13	0.13	[−0.13, 0.39]
Individualism	−0.01	0.04	−0.02	[−0.10, 0.07]
Collectivism	0.10	0.05	0.12	[0.03, 0.21]
Anxious Attachment	0.01	0.08	0.00	[−0.16, 0.17]
Avoidant Attachment	0.03	0.09	0.02	[−0.16, 0.2]

*Notes: b*, Unstandardized regression coefficient; *SE*, Standard error; *β*, standardized regression coefficient; CI, Confidence interval of standardized regression coefficient. ^a^ Women = 1, men = 2, * *p* < 0.01, ** *p* < 0.001.

**Table 4:** Regression Coefficients of Study Variables on External—Family Locus-of-Hope.

Variable	*b*	*SE*	*β*	95% CI
(Intercept)	−4.93	1.91	0.00	[−3.76, 3.76]
Gender^ a^	0.10	0.34	0.01	[−0.65, 0.68]
Self-esteem	0.06	0.03	0.08	[0.01, 0.15]
Relational self-esteem	0.40 **	0.06	0.34	[0.23, 0.46]
Individualism	−0.01	0.02	−0.02	[−0.06, 0.02]
Collectivism	0.15 **	0.02	0.33	[0.29, 0.38]
Anxious Attachment	−0.03	0.04	−0.03	[−0.11, 0.04]
Avoidant Attachment	0.02	0.04	0.03	[−0.05, 0.11]

*Notes: b*, Unstandardized regression coefficient; *SE*, Standard error; *β*, standardized regression coefficient; CI, Confidence interval of standardized regression coefficient. ^a^ Women = 1, men = 2, * *p* < 0.01, ** *p* < 0.001.

**Table 5:** Regression Coefficients of Study Variables on External—Peer Locus-of-Hope.

Variable	*b*	*SE*	*β*	95% CI
(Intercept)	5.04 *	1.95	0.00	[−3.83, 3.83]
Gender^ a^	−0.44	0.34	−0.06	[−0.73, 0.62]
Self-esteem	0.06	0.04	0.08	[0.01, 0.15]
Relational self-esteem	0.16 *	0.06	0.15	[0.03, 0.26]
Individualism	−0.04	0.02	−0.10	[−0.14, −0.07]
Collectivism	0.15 **	0.02	0.35	[0.30, 0.39]
Anxious Attachment	−0.03	0.04	−0.05	[−0.12, 0.02]
Avoidant Attachment	−0.12 *	0.04	−0.16	[−0.24, −0.07]

*Notes: b*, Unstandardized regression coefficient; *SE*, Standard error; *β*, standardized regression coefficient; CI, Confidence interval of standardized regression coefficient. ^a^ Women = 1, men = 2, * *p* < 0.01, ** *p* < 0.001.

## Data Availability

Data are available from any of the authors.

## Appendix A

**Table A1:** Cultural Identifications Accounting for the Largest Percentages of Participants.

Identified Culture	Amount of Sample (%)
Canadian	11.8
Canadian and Chinese	1.8
Canadian and Italian	1.3
Caucasian (white, western, European)	7.7
Chinese	19.4
Indian	2.6
South Asian	1.3
Missing or inconclusive	16.9

Note: This table shows a small sample of participants’ responses to a fill-in-the-blank question on general cultural identification and the percentages of participants who identified with such cultures. Therefore, the identified cultures do not reflect any specific or predefined criteria of cultural categorization.
